# Retained Glass Fragment in the Cervical Spinal Canal in a Patient with Acute Transverse Myelitis: A Case Report and Literature Review

**DOI:** 10.1155/2018/5129513

**Published:** 2018-05-31

**Authors:** Simonas Jesmanas, Kristina Norvainytė, Rymantė Gleiznienė, Algirdas Mačionis

**Affiliations:** ^1^Faculty of Medicine, Medical Academy, Lithuanian University of Health Sciences, Kaunas, Lithuania; ^2^Department of Radiology, Medical Academy, Lithuanian University of Health Sciences, Kaunas, Lithuania; ^3^Department of Neurology, Medical Academy, Lithuanian University of Health Sciences, Kaunas, Lithuania

## Abstract

A 50-year-old male presented with a one-day history of right leg weakness, numbness, and urinary retention. Weakness was present for two weeks but worsened significantly during the last 24 hours. On the right there was sensory loss in the leg and below the Th8 dermatome. On the left there was sensory loss below the Th10 dermatome and distal loss of temperature sensation. Past medical history revealed a cervical trauma 30 years ago when a glass chip lodged into the left side of the neck. The patient did not seek medical attention after removing it himself. No neurological symptoms followed the incident. No cervical manipulation or other physical trauma occurred before current symptom onset. Magnetic resonance (MR) imaging showed features consistent with myelitis at the level of C4–Th3. At the level of C6–C7, a T1 and T2 hypointense lesion was noted. On computed tomography, this lesion was hyperdense and occupied the spinal canal and the left intervertebral foramen. It was deemed to be a glass fragment. Surgical removal was withheld because the fragment was clinically silent for 30 years, the risk of surgical removal would outweigh the benefits and the patient did not prefer surgical treatment. Acute demyelinating transverse myelitis was diagnosed and treated with methylprednisolone. 10 months later MR features of myelitis resolved and the patient's neurological condition improved. Our case shows that foreign bodies in the cervical spinal canal can remain asymptomatic for up to 30 years. In the case of a long asymptomatic retention period the need for surgical removal of a foreign body must be carefully evaluated, taking into account the probability that a foreign body is the cause of current symptoms, risk of a foreign body causing damage in the future, risk of damage to the spinal cord during removal, and probability of therapeutic success.

## 1. Introduction

The vertebral column does not provide perfect protection to the spinal cord. Multiple openings, most notably the intervertebral foramina, exist for passage of structures to and from the spinal canal. Those same openings can serve as pathways by which foreign bodies can enter and damage the contents of the spinal canal. Various rarely encountered accidental injuries by metallic, wooden, and other objects or medical intervention can cause traumatic penetrating nonmissile spinal cord injury [1–3]. Spinal cord trauma most often presents with either complete or incomplete tetraplegia or paraplegia with or without accompanying sensory, autonomic (bowel and bladder function) disturbance [1]. Acute demyelinating transverse myelitis can also present with a similar clinical picture, particularly with a sensory level, although there usually will not be a history of trauma and the course is likely to be acute-to-subacute, reaching its peak over several weeks [4]. We present a rare case of a clinically silent glass fragment impaction into the cervical spinal canal which went undiscovered for several decades until an episode of acute demyelinating transverse myelitis and provide a literature review.

## 2. Clinical Case

A 50-year-old male patient presented with a one-day history of right leg weakness, numbness, and urinary retention. There was mild back pain and right leg weakness for two weeks which worsened significantly during the last 24 hours. On neurological examination the patient‘s right leg was weaker than the left (2/5 and 4/5 on Lovett test, respectively), the patellar reflex was exaggerated, and Babinski sign was positive bilaterally. Also, on the right side, there was sensory loss in the leg and below the Th8 dermatome. On the left, there was sensory loss below Th10 dermatome and distal loss of temperature sensation.

Past medical history revealed a cervical trauma which occurred 30 years ago when a glass chip lodged into the left side of the patient's neck. The patient removed the visible glass shard from his neck and did not seek medical attention; therefore no clinical and radiological investigations were carried out. No neurological symptoms followed this incident.

**Figure 1:**
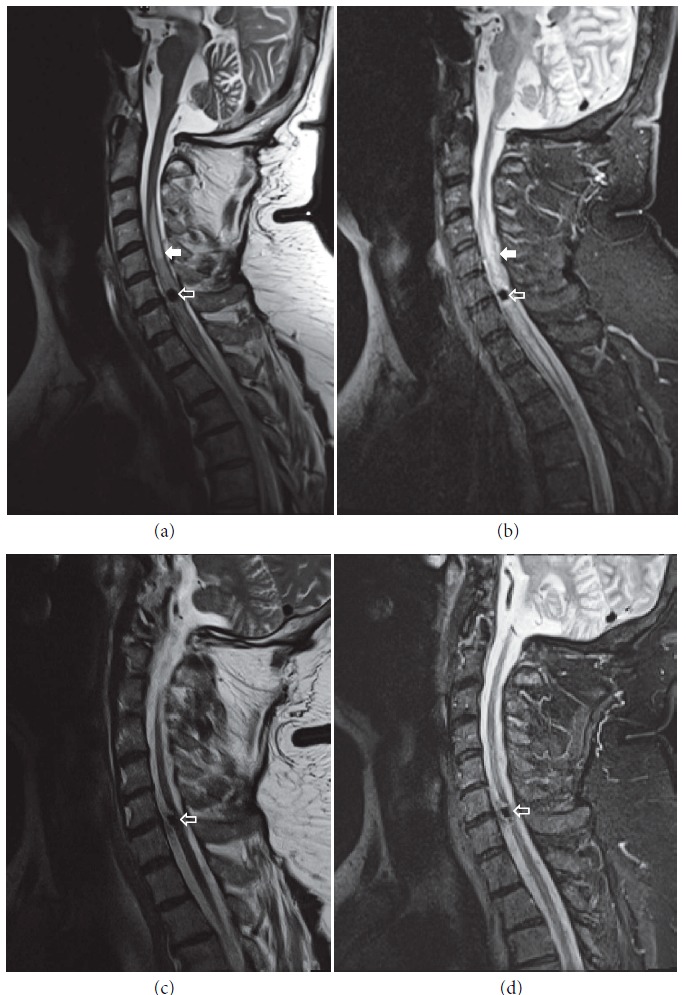
**MRI of the cervical spine: initial MRI at symptom onset**: (a) sagittal T2W/TSE (TR 3800 ms, TE 108 ms); (b) sagittal T2W/STIR (TR 4000 ms, TE 39 ms). Images show a hypointense oblong lesion in the spinal cord at the level of C6-C7 (empty arrows). Above and below the lesion the spinal cord shows hyperintense signal consistent with myelitis (filled arrows).** Follow-up MRI 10 months later**: (c) sagittal T2W/TSE (TR 3800 ms, TE 108 ms) and (d) sagittal T2W/STIR (TR 4000 ms, TE 39 ms). Follow-up images show normal spinal cord appearance following the resolution of myelitis and the same hypointense glass lesion (empty arrows).

**Figure 2:**
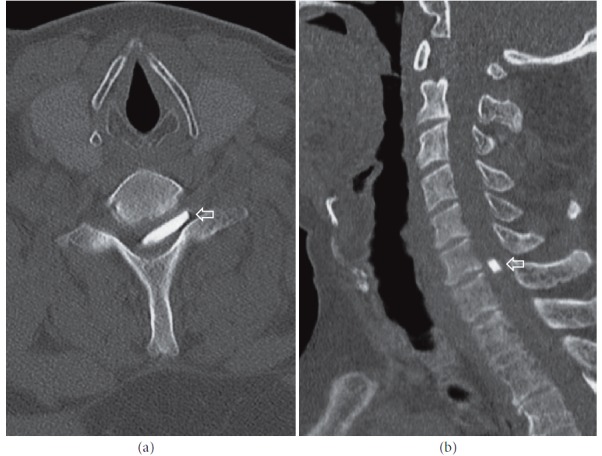
**CT of the cervical spine: **bone windows, axial (a) and sagittal reconstruction (b) showing a hyperdense oblong lesion at the level of C6-C7 in the left intervertebral foramen and spinal canal (arrows).

Computed tomography (CT) of the lumbosacral and thoracic regions showed a mild convexity of the L5-S1 intervertebral disc with no other clinically significant findings (“Siemens SOMATOM Emotion 6”) (images not shown). To further explore the possible causes of the patient's symptoms, magnetic resonance imaging (MRI) of the C1–L2 segments was performed (“Siemens MAGNETOM Avanto 1.5 T”). At the level of C4–Th3 the spinal cord was thickened and hyperintense on T2W images, features consistent with myelitis; however there was no appreciable contrast uptake (Figures 1(a) and 1(b)). At the level of C6–C7 an oblong (1.6 x 0.4 cm), T1 and T2 hypointense lesion was found (Figure 1). Because a foreign body was suspected, CT scan of the C1–Th3 levels was performed and demonstrated a hyperdense lesion occupying the spinal canal and the left intervertebral foramen (Figure 2).

**Figure 3:**
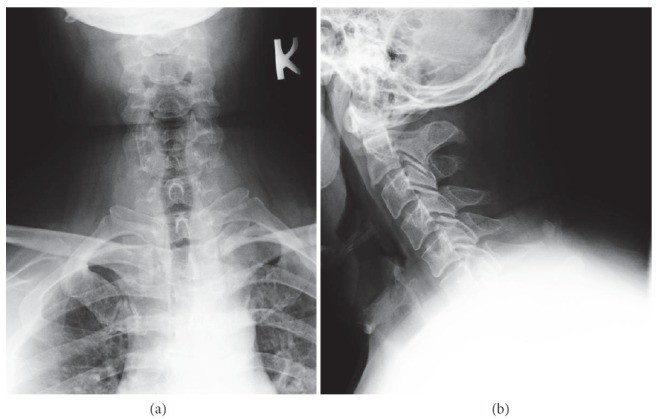
**X-ray of the cervical spine.** The foreign body is not visible in posteroanterior (a) and lateral (b) views.

Combining the CT and MRI results with the past medical history of an old injury with a glass fragment, it was determined that the lesion represented a glass foreign body in the spinal canal. The patient also had an X-ray of the cervical spine but the foreign body could not be visualized, most likely due to being located at the level of C6-C7, where it was obscured by the surrounding structures (Figure 3).

Taking into account the clinical picture, an extensive period of time between the trauma and current presentation, and MR imaging findings, an acute demyelinating episode rather than traumatic spinal cord injury was suspected. Further diagnostic work-up would typically have included a lumbar puncture to identify oligoclonal bands, cells, and protein, but it was contraindicated due to the risk of disturbing the foreign body and causing it to migrate upon a sudden decrease in pressure during puncture. Serum Aquaporin-4-specific antibodies could not be performed at the time and were planned for a later time.

The patient fulfilled the inclusion criteria for acute transverse myelitis: bilateral and not necessarily symmetrical sensory, motor, and autonomic spinal cord dysfunction, a clear sensory level, peak of symptoms within 4 hours and 21 days after onset of symptoms, and exclusion of other causes (neoplastic, vascular, and compressive) [5]. Compressive cause was excluded because the spinal cord pathology seen on MRI extends far from the location of the glass shard, which would be unlikely given the size of the foreign object and its possible effect upon the spinal cord if it migrated within the spinal canal. Thus, because the glass fragment lay dormant for the last 30 years, it was deemed not to be the direct cause of the patients' symptoms.

Treatment with methylprednisolone 500 mg intravenously daily for 6 days was initiated.

After consultation with the neurosurgeons it was decided not to remove the foreign body from the spinal canal, because the risks of surgery would outweigh the benefits. At the time of consultation, the patient was already showing improvement on medical management. Given that the situation was not hyperacute, the symptoms were better explained by the inflammatory and demyelinating reaction within the spinal cord rather than direct contact with the foreign body. Also, it could not be guaranteed that removing the glass shard would result in symptomatic improvement. Upon removal of the foreign body some diffuse bleeding would be expected, which combined with the already inflamed spinal cord parenchyma would likely further compromise the spinal cord, potentially causing vascular complications and myelomalacia, all of which would further decrease the chance of clinical improvement. Risk of general surgical complications (postoperative infection, bleeding, and thromboembolism) further argued against surgical treatment. An absolute indication for surgical treatment would be an infectious complication of the foreign body, which was not present. The patient agreed with the treating physicians that surgery would not be the best option and did not want the operation. If current medical treatment would have proven unsuccessful, and the patient's clinical condition worsened, surgery would have been indicated.

During the course of treatment the patient's condition improved. Sensory loss diminished, and the right leg strength improved to 4/5 on Lovett test, but urinary retention remained. Intermittent catheterization was prescribed.

The patient returned for a follow-up visit 10 months later with a stable and improved neurological state. Lower limb strength was 3/5 proximally and 4/5 distally, with positive bilateral Babinski sign. Minimal intermittent urinary retention remained but did not significantly impair the patients' quality of life. The patient resumed his activities of daily living and continues to work as a security guard. Follow-up MRI of the cervical spine shows the same oblong hypointense object and normal spinal cord after the resolution of myelitis (Figures 1(c) and 1(d)).

Further follow-up is scheduled every 6 to 12 months, with an outpatient brain MRI to identify any other demyelinating lesions that may be present in case this episode was part of neuromyelitis optica (NMO), acute demyelinating encephalomyelitis (ADEM), or multiple sclerosis (MS).

## 3. Discussion and Literature Review

### 3.1. Glass Foreign Bodies and Delayed Symptom Onset

In total we found 17 cases of glass spinal injuries reported in the literature [6–22].

Of particular interest to our case is the sometimes observed significant time lag between injury and symptom onset or complete absence of neurological symptoms after apparently substantial disruption of the spinal cord by a foreign body. 5 out of 17 cases presented months or years after the injury, ranging from 3.5 months to 21 years [8, 11, 16, 18, 19]. In our described case, the time until discovery of a glass fragment was greater than in any of the studies reported in the literature. Vadasz et al. noted that the delay between the initial injury and symptom onset can be either subacute, due to CSF leak, meningitis, or abscess, or chronic, due to myelomalacia and spinal cyst formation [12].

While our patient had the foreign body for several decades, clinical and imaging findings were most consistent with an acute demyelinating myelitis. Some authors have reasoned that slow migration of the foreign body can cause spinal cord damage to manifest [11, 18, 19], but we think this was unlikely in our case. The small amount of kinetic energy arising from a slow migration of a relatively small object would be unlikely to cause such extensive spinal cord involvement seen on the spinal MRI. Indeed, in such a scenario the MRI appearance should resemble that of localized compressive myelopathy due to intervertebral disc pathology or chronic localized spinal stenosis of other etiologies.

Acute transverse myelitis is usually acute or subacute in onset, and neurological symptoms tend to worsen and reach their peak after a few weeks [4]. The name “transverse” myelitis is rather historical in nature, because demyelination does not always completely encompass the spinal cord transversely. However, the presence of a sensory level and an acute-to-subacute course are crucial, which were present in our patient. It can be further divided into complete or partial, with the latter including hemicord (Brown-Sequard), central cord, dorsal column, or specific tract disruption [4]. Longitudinally extensive transverse myelitis, which involves more than 3 segments, was detected in our case. The symptoms were mostly localized to the right spinal cord producing a mostly hemicord clinical picture, with at least some central and contralateral cord involvement producing a mixed symptomatology. The glass foreign body hypothetically could have been a predisposing factor for an autoimmune demyelinating process of the spinal cord; however, we cannot confidently exclude a simple coincidence. Therefore, in our view, the foreign body has remained asymptomatic despite the patients' current condition.

It is interesting to note that in cases when the cervical spine was injured, the symptoms were logically explained by the location: quadriparesis and anesthesia or hypoesthesia [7, 13, 21], hemiparesis on the left and hypoesthesia with spared deep sensations on the right [8, 10], and dysesthesia in the arms [16, 18]. However, in our case, the absence of any upper extremity symptoms is surprising, because the glass shard is at the level of the segments supplying the brachial plexus and lodged into the C6/C7 intervertebral foramen, where it most likely should have damaged the corresponding nerve root. One possible explanation of the absence of nerve root symptoms would be that this patient had anomalous nerve root origin, whereby no root was present in the C6/C7 intervertebral foramen; however such anomalies are more common in the lumbosacral region [23].

### 3.2. Removal of Retained Glass Foreign Bodies from the Spinal Canal

The management of glass fragments in the described cases was always surgical (laminectomy, removal of the fragments, and dural closure) if the patient presented to the hospital immediately after injury and exhibited signs of neurological damage and the glass fragment was successfully visualized with imaging [7–10, 13, 15, 20–22]. One patient died due to cardiopulmonary insufficiency [7], while others survived, but some exhibited varying degrees of neurological dysfunction [10, 13, 20, 21].

Some authors reasoned that delayed presentation in the presence of a foreign body is usually precipitated by a small injury, which disturbs the foreign body, causes it to move to a greater or lesser extent and triggers the onset of symptoms by affecting spinal cord function [11, 18, 19]. The decision reached by Oertel et al. to withhold surgical removal of the glass fragment after it was lodged for 12 years seems to support this conclusion, as the authors noted that fibrous tissue had already formed and seemed to be protecting surrounding the spinal cord and nerve roots [16]. Symptoms probably appeared because of progressively narrowing vertebral canal due to degeneration [16]. Removal of the fragment would disrupt the protective tissue and may in itself cause neurological damage; however the possibility of the fragment being displaced and causing symptoms on its own cannot be absolutely excluded [16]. In other cases with a delayed presentation, the surgeons chose to operate and remove the fragments due to severe neurological impairment and achieved therapeutic success [8, 19]. In our case surgical treatment was withheld for the reasons outlined in the case description.

Akcakaya et al. emphasized that usually foreign body removal is indicated if there is a possibility of toxicity, for example, if it is composed of lead or copper [18]. When foreign bodies remain in the vertebral canal for a long period of time, they can slowly degrade or corrode and cause sustained tissue irritation, which can lead to chronic inflammation and progressive damage to nerve roots or spinal cord itself, causing pain or a delayed neurological deficit [24]. While we do not have any evidence to support our hypothesis, we think it is plausible that the glass fragment could have played some part in predisposing the patient to develop acute demyelinating transverse myelitis, by causing either localized chronic irritation or immunological disturbance.

### 3.3. Imaging

Glass of at least 2 mm in size should always be visible on X-rays due to its higher density and effective atomic number when compared with soft tissues [3, 25]. However, as our case illustrates, if there is sufficient obstruction of the view by surrounding radiopaque structures, it may be obscured. On CT it is always hyperdense. MR signal is derived from hydrogen protons, but glass has no hydrogen atoms; therefore it produces no signal and is dark on both T1 and T2. Even if MR does not provide good visualization of the foreign body itself, it can be useful to show the reactive changes of the surrounding tissues, lacerations, signs of infection, inflammation, and demyelination [26].

MR features of acute spinal cord injury (SCI) include smooth enlargement of the cord contour, hyperintensity on T2W and hypointensity on T1W, and hemorrhage [27]. In cases of subacute SCI a progressive decrease in overall spinal cord area above the lesion level can be seen on MRI [28]. Eventually, this decrease may lead to the spinal cord atrophy, the most common finding in patients imaged more than 20 years after the initial trauma [27]. In a patient with history of previous spinal injury and new onset of neurological deficits, there is a possibility of posttraumatic syringomyelia [29]. Neither atrophy nor syringomyelia was present in our patient. Myelomalacia demonstrates similar findings to myelitis [27] and would be more likely without the acute-to-subacute clinical picture. Longitudinally extensive transverse myelitis encompasses more than 3 segments and exhibits T2W hyperintensity and sometimes T1W hypointensity, swelling, and none or variable enhancement [30], and it was the best fit in our case.

Idiopathic demyelinating transverse myelitis should be distinguished from transverse myelitis occurring with other demyelinating conditions, specifically neuromyelitis optica (NMO), multiple sclerosis (MS), and acute demyelinating encephalomyelitis (ADEM). MS lesions are usually multiple, located in the peripheral spinal cord, and less extensive, exhibiting a distribution in space and time [31]. Follow-up imaging of the spinal cord and MR of the brain are important to look for widely distributed or new demyelinating lesions which would support the diagnosis of MS. Immunologic testing for aquaporin-4 antibodies and visually evoked potentials are also important in ruling out NMO [31]. Our patient will continue to be followed and evaluated for signs and symptoms of ADEM, NMO, and MS.

## 4. Conclusion

In conclusion, we report a very rare case of a 50-year-old male with a glass shard in the cervical spinal canal which has remained clinically silent for 30 years until an episode of acute demyelinating transverse myelitis. Physicians must keep in mind that foreign bodies in the spinal canal can produce no neurological symptoms for up to 30 years. In the case of a long asymptomatic retention period the need for surgical removal of a foreign body must be carefully evaluated, taking into account the estimated probability that a foreign body is the cause of current symptoms or just an incidental finding, risk of a foreign body causing damage in the future, risk of further damage to the spinal cord during removal, and probability of therapeutic success.
